# The Role of circRNAs in the Diagnosis of Colorectal Cancer: A Meta-Analysis

**DOI:** 10.3389/fmed.2021.766208

**Published:** 2021-11-19

**Authors:** Ru-Dong Li, Min Guan, Zhe Zhou, Shu-Xiao Dong, Qian Liu

**Affiliations:** ^1^Department of Gastrointestinal Surgery, Shandong Provincial Third Hospital, Cheeloo College of Medicine, Shandong University, Jinan, China; ^2^Department of Respiratory Medicine, Shandong Provincial Third Hospital, Cheeloo College of Medicine, Shandong University, Jinan, China

**Keywords:** circRNA, colorectal cancer, biomarker, diagnostic power, meta-analysis

## Abstract

**Background:** A novel category of non-coding circular RNAs (circRNAs) has been found to be dysregulated in colorectal cancer (CRC) and significantly contribute to its progression. However, the feasibility of using circRNA as a diagnostic biomarker for CRC remains to be elucidated. Herein, we aimed to comprehensively collect and analyze evidence regarding the potential application of circRNAs as diagnostic indicators for CRC.

**Methods:** A comprehensive retrieval of relevant studies dating from January, 2015 to December 2020, was carried out in PubMed, Cochrane Library, and Web of Science. Data regarding the diagnostic accuracy of circRNA for CRC, including sensitivity, specificity, positive likelihood ratio (PLR), negative likelihood ratio (NLR), diagnostic odds ratio (DOR), and area under the curve (AUC), were obtained from the included studies. Quality assessment of diagnostic accuracy studies (QUADAS-2) was used to assess the methodological quality of each study. Statistical analysis was performed using STAT and RevMan software.

**Results:** Eighteen studies, involving a total of 2021 individuals, were included in the present meta-analysis. The specimens examined included tissue, serum, and plasma. The pooled sensitivity, specificity, DOR, PLR, NLR, and AUC, with a 95% confidence interval (CI), of circRNAs in the diagnosis of CRC were 0.78 (0.71–0.83), 0.73 (0.68–0.78), 9.68 (6.76–13.85), 2.92 (2.45–3.50), 0.30 (0.23–0.39), and 0.81 (0.78–0.85), respectively. Subgroup analysis showed that the upregulated circRNAs in the tissue or plasma possessed relatively higher diagnostic values for CRC than the downregulated circRNAs. There was no significant difference between the tissue-derived and non-tissue-derived circRNA subgroups.

**Conclusion:** circRNA may be used as a diagnostic biomarker for CRC because of its relatively high diagnostic accuracy in distinguishing CRC patients from normal controls. Further prospective studies are needed to identify more representative circRNAs as diagnostic markers for CRC.

## Introduction

Colorectal cancer (CRC) is one of the most common malignancies and a leading cause of cancer mortality worldwide; the survival rate of patients with advanced CRC is quite low, thus necessitating its early diagnosis as well as the development of improved therapeutic strategies to reduce the death rate (1, 2). Currently, histological evaluation of biopsy is the gold standard for CRC detection. However, definitive diagnosis requires endoscopic evaluation, which is time-consuming, invasive, and involves a series of complications (3). Hence, despite its relatively high sensitivity and specificity, its use for the early diagnosis of CRC is still limited in the clinic. Though several clinical biomarkers, such as CA199 and CEA, have been used for tumor diagnosis, the biomarker-generated clues are not reliable for diagnosing CRC owing to their low sensitivity and specificity (4). Therefore, there is a desperate need for identifying novel feasible biomarkers for CRC diagnosis.

Circular RNAs (circRNAs), a type of non-coding RNA first identified in RNA viruses, is generated by linking the 3′ and 5′ ends with covalent bonds (5, 6). After years of being neglected, the improvement in experimental methods confirmed their presence in the human body, opening up a way to meaningful research regarding the role of circRNAs in human pathophysiological processes (7). Several mechanisms are utilized by circRNAs to exert their regulatory effects on biological processes. The presence of abundant binding sites for miRNAs confers circRNAs the potential to act as a sponge to absorb miRNA (8). In addition, circRNAs can also interact with proteins to regulate their activity (9). Recent studies have suggested the regulatory role of circRNA in tumor progression, such as in tumor proliferation (10), immune evasion (11), apoptosis resistance (12), invasion, and migration (13). In addition, they are stably expressed in the cells because they lack open linear tail, which protects them from degradation by exonucleases (14). The correlation between circRNAs and the stage of CRC suggests their potential contribution to tumor progression, which indicates that circRNAs may serve as novel biomarkers for CRC.

In this study, we conducted a meta-analysis to assess the feasibility of using circRNAs as biomarkers for the early diagnosis of CRC according to their sensitivity and specificity.

## Methods

### Search Strategy

Retrieval of relevant studies, published in English between 2015 and 2020, was conducted in PubMed, Cochrane Library, and Web of science online databases using the following key words: “circular RNA” or “circRNA” and “colorectal,” “cancer,” or “malignant tumor.” Eligible studies were screened by two investigators (M. Guan and Z. Zhou) by going through the title, abstract, and full text of each article. Disagreements were resolved by reaching a consensus after detailed discussion with a third investigator (Ru-Dong Li). The data of the included articles were then collected.

### Inclusion and Exclusion Criteria

Articles meeting the following criteria were included:

(1) the diagnostic accuracy of circRNAs as a biomarker for CRC was assessed;
(2) confirmation of CRC by histological examination;
(3) true-positive (TP), false-positive (FP), false-negative (FN), and true-negative (TN) values should be mentioned in the article or can be derived from the article,
(4) should include experimental and control groups; and
(5) the total number of samples, sensitivity, specificity, and area under the curve (AUC) should be available in the article.

Articles meeting the following criteria were excluded:

(1) research subject was not human;
(2) reviews, conference abstract, commentary articles or response;
(3) articles without sufficient data; and
(4) articles examining the accuracy of prognosis prediction.

### Data Extraction

Data from the selected articles, including details of the first author, publication year, specimen source, reference gene, circRNA testing method, total number of samples (experimental and control), sensitivity, specificity, and AUC, were collected by the two investigators independently by reading the full text of each article. The TP, FP, FN, and TN values were obtained from the articles or were calculated based on the total number of samples, sensitivity, and specificity values obtained from the articles.

### Quality Assessment

The included studies were independently assessed by two reviewers using QUADAS-2 (15).

### Statistical Analysis

The analytical software STATA (version 15), Meta-disc (version 1.4), and RevMan (version 5.3) were used to perform statistical analyses. The diagnostic values, including sensitivity, specificity, diagnostic odds ratio (DOR), positive likelihood ratio (PLR), negative likelihood ratio (NLR), and AUC of the circRNAs associated with CRC diagnosis, were analyzed and their 95% CIs were plotted with a two-sided *p* < 0.05, which was considered statistically significant. A random-effects model was used for significant heterogeneity (I^2^ > 50%), and a fixed-effects model was used for minimal heterogeneity (I^2^ < 50%). The contribution of the threshold effect to the heterogeneity of the included studies was quantified using Spearman's correlation analysis. The non-threshold effect was evaluated using the chi-squared test and I^2^ statistics, and I^2^ > 50% (*p* < 0.1) represented the existence of heterogeneity caused by a non-threshold effect. Meta-regression was conducted to determine the possible origin of heterogeneity caused by the non-threshold effect. Subgroup analysis was carried out to assess the superiority of combination strategy over individual circRNAs, in either tissue or plasma, as the diagnostic biomarker for CRC.

## Results

### Study Selection and the Characteristics of the Included Studies

**Figure 1:**
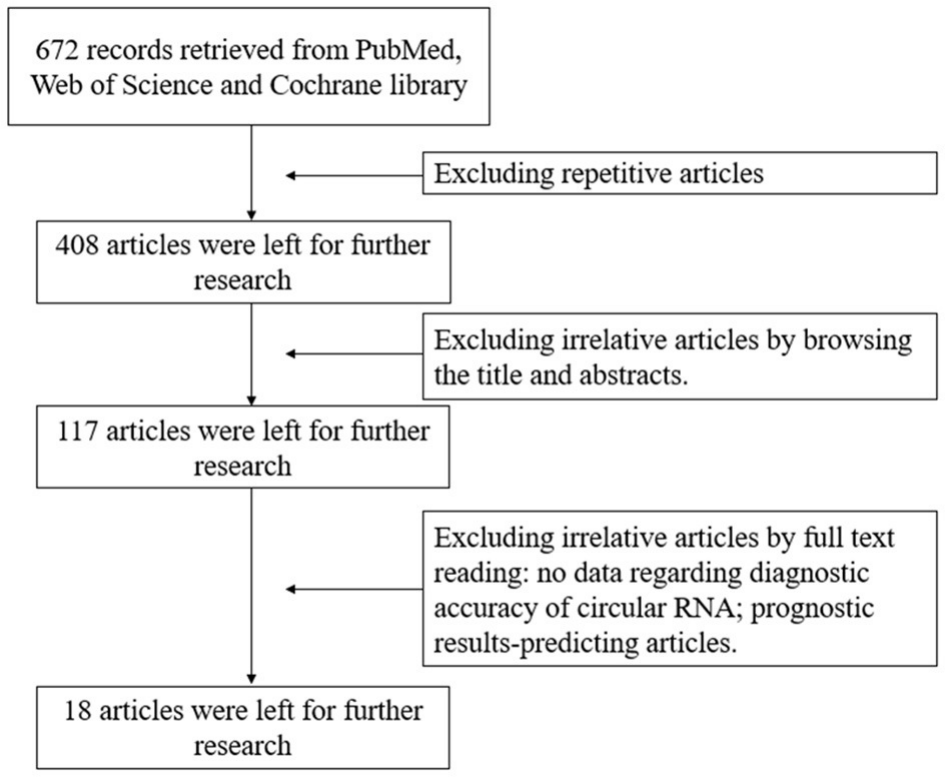
Flowchart of study selection process. The meta-analysis identified 18 eligible studies that used circular RNAs as diagnostic biomarkers for colorectal cancer.

Based on the inclusion and exclusion criteria, 18 articles (16–32) involving 2021 individuals were included in our meta-analysis after filtering 672 articles retrieved from the PubMed, Cochrane Library, and Web of Science online databases. A flowchart describing the detailed retrieval strategy is presented in Figure 1.

The included studies aimed to assess the diagnostic value of circRNAs for CRC and were published in English between January, 2015 and December, 2020. All of the included articles used GAPDH as an endogenous reference. These articles assessed the expression value of circRNA in the tissue, serum, or plasma, for diagnosing CRC. Among them, eight and 10 studies assessed the diagnostic values of upregulated and downregulated circRNAs, respectively, while 12 and six studies assessed the diagnostic values of tissue-derived and non-tissue-derived circRNAs, respectively. The AUC of circRNA as a diagnostic biomarker for CRC ranged from 0.616 to 0.884. The characteristics of the included articles are summarized in Table 1.

**Table 1:** Characteristics of the included studies.

**References**	**Year**	**circRNAs profiles**	**Sample size (case/control)**	**Specimen source**	**Expression level**	**Reference gene**	**Sensitivity**	**Specificity**	**AUC**
Hsiao et al. (19)	2017	has_circ_0001313	131/76	tissues	up-regulated	GAPDH	0.931	0.738	0.884
Sadeghi et al. (18)	2020	hsa_circ_0060927	83/83	tissues	up-regulated	GAPDH	0.68	0.83	0.78
Zhang et al. (24)	2018	hsa_circ_0007534	112/46	plasma	up-regulated	GAPDH	0.92	0.522	0.78
Li et al. (31)	2019	hsa_circ_0006990	60/43	plasma	up-regulated	GAPDH	0.7	0.651	0.724
Lin et al. (17)	2019	circ-CCDC66/circ-STIL /circ-ABCC1	45/61	plasma	up-regulated	GAPDH	0.644	0.852	0.78
Barbagallo et al. (20)	2018	hsa_circ_0000284	20/20	serum	up-regulated	GAPDH	0.71	0.8	0.771
Pan et al. (21)	2019	hsa-circ-0004771	110/35	serum	up-regulated	GAPDH	0.809	0.829	0.88
Xie et al. (16)	2020	circ-PNN	58/58	serum	up-regulated	GAPDH	0.897	0.69	0.826
Wang et al. (29)	2015	hsa_circ_001988	31/31	tissues	down-regulated	GAPDH	0.68	0.74	0.788
Zhuo et al. (27)	2017	hsa_circ_0003906	122/40	tissues	down-regulated	GAPDH	0.803	0.725	0.818
Zhang et al. (22)	2017	hsa_circ_103809	170/170	tissues	down-regulated	GAPDH	0.664	0.695	0.699
Zhang et al. (22)	2017	hsa_circ_104700	170/170	tissues	down-regulated	GAPDH	0.682	0.529	0.616
Wang et al. (23)	2018	hsa_circ_0014717	46/46	tissues	down-regulated	GAPDH	0.432	0.87	0.683
Wang et al. (30)	2018	hsa_circ_0000567	102/102	tissues	down-regulated	GAPDH	0.833	0.765	0.865
Li et al. (28)	2018	hsa_circ_0000711	101/101	tissues	down-regulated	GAPDH	0.91	0.58	0.81
Ji et al. (25)	2018	hsa_circ_0001649	64/64	tissues	down-regulated	GAPDH	0.828	0.781	0.857
Ruan et al. (32)	2019	hsa_circ_0002138	35/35	tissues	down-regulated	GAPDH	0.629	0.743	0.725
Ge et al. (26)	2019	hsa_circ_0142527	41/41	tissues	down-regulated	GAPDH	0.829	0.805	0.818

### Quality Assessment

**Figure 2:**
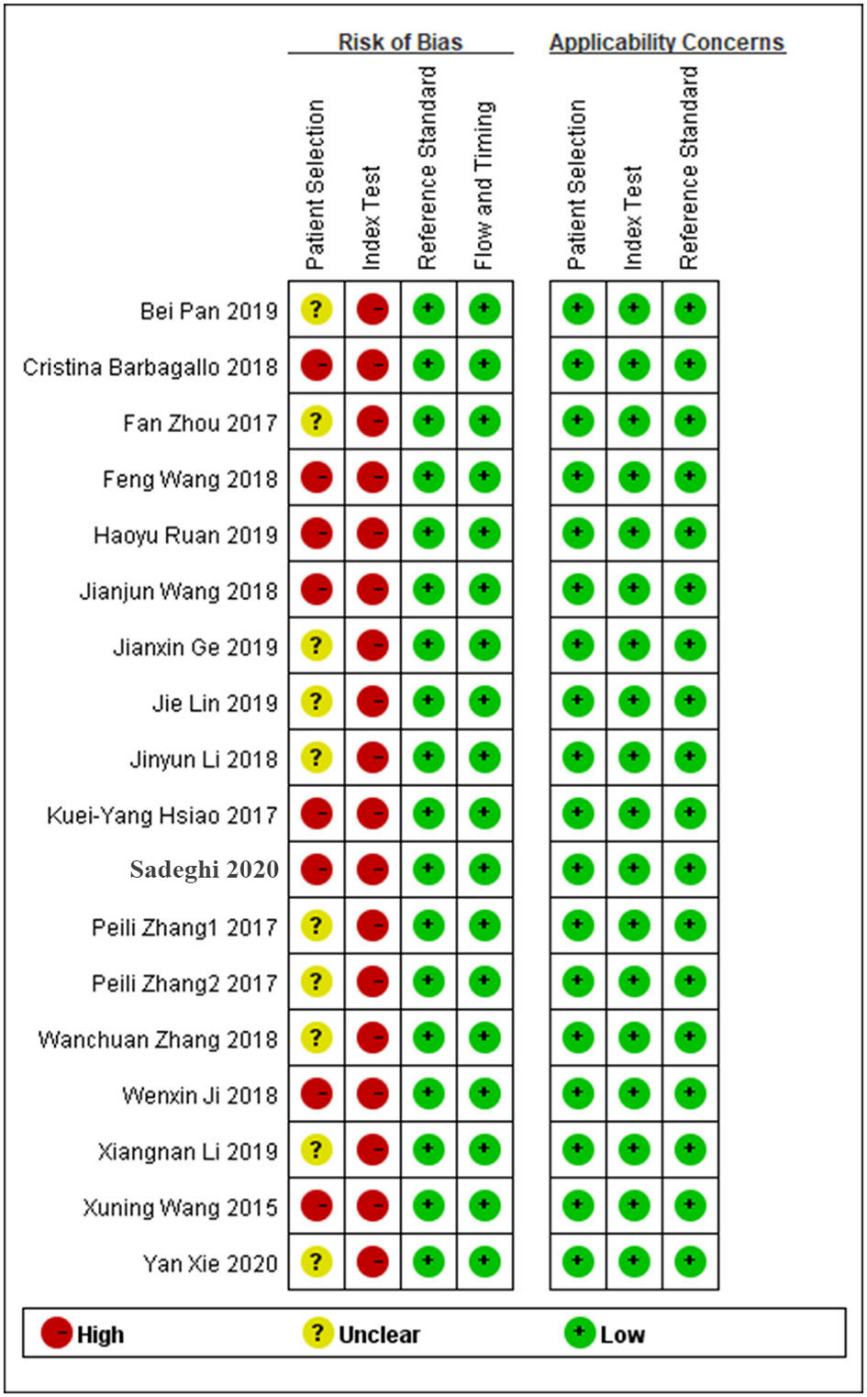
Methodological quality summary.

The quality of the enrolled studies was assessed using the QUADAS-2 evaluation tool, and the results are shown in Figure 2. The quality of the enrolled articles varied from moderate to high, suggesting a relatively reliable foundation for our analysis.

### Meta-Analysis of the Diagnostic Power of circRNA

**Figure 3:**
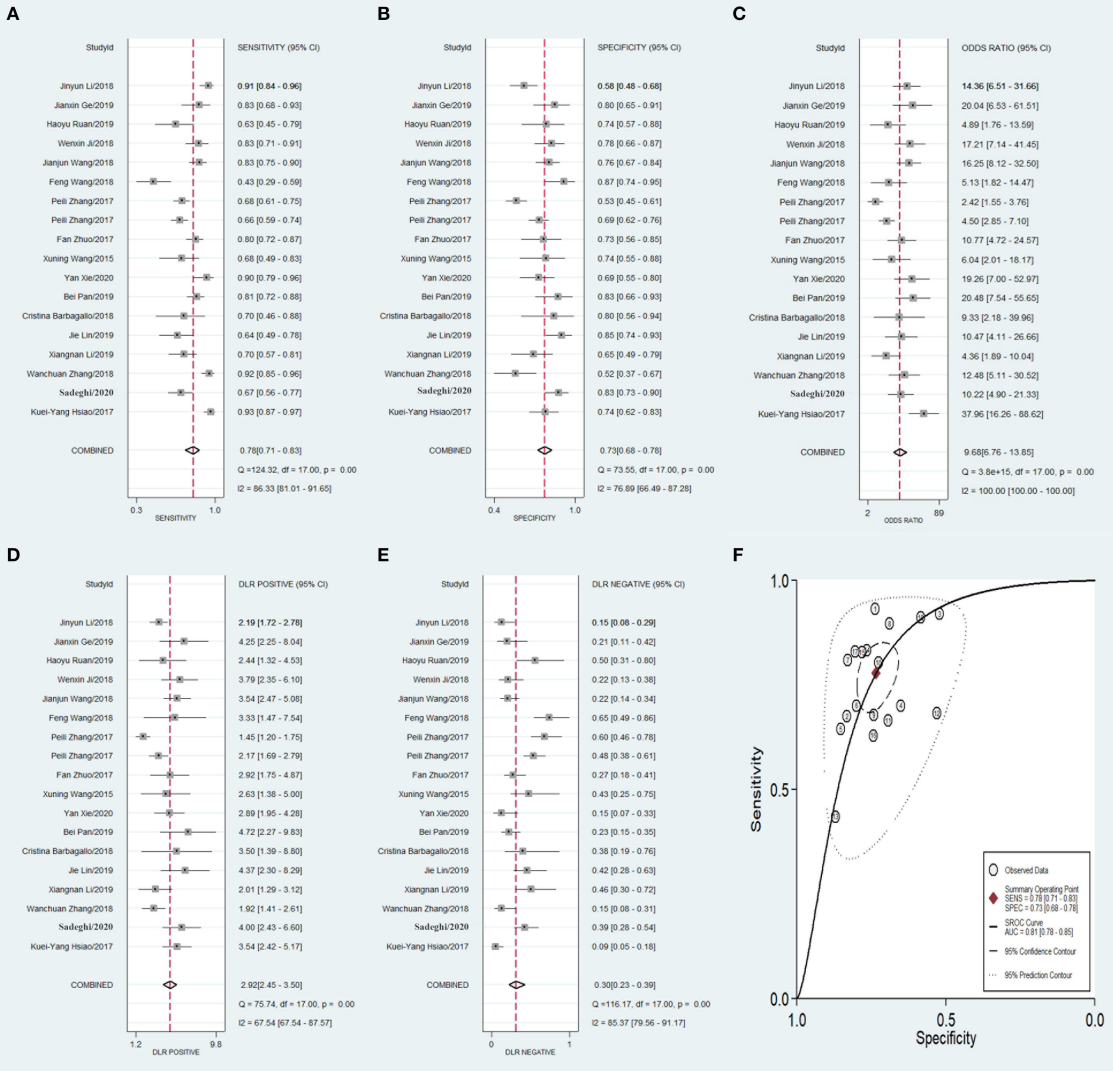
Forest plots of pooled diagnostic power of circRNA as a biomarker for colorectal cancer among 18 articles, **(A)** Sensitivity, **(B)** Specificity; **(C)** DOR; **(D)** PLR; **(E)** NLR and **(F)** AUC. DOR, diagnostics odds ratio; PLR, positive likelihood ratio; NLR, negative likelihood ratio; AUC, the area under receiver operating characteristics curve.

**Figure 4:**
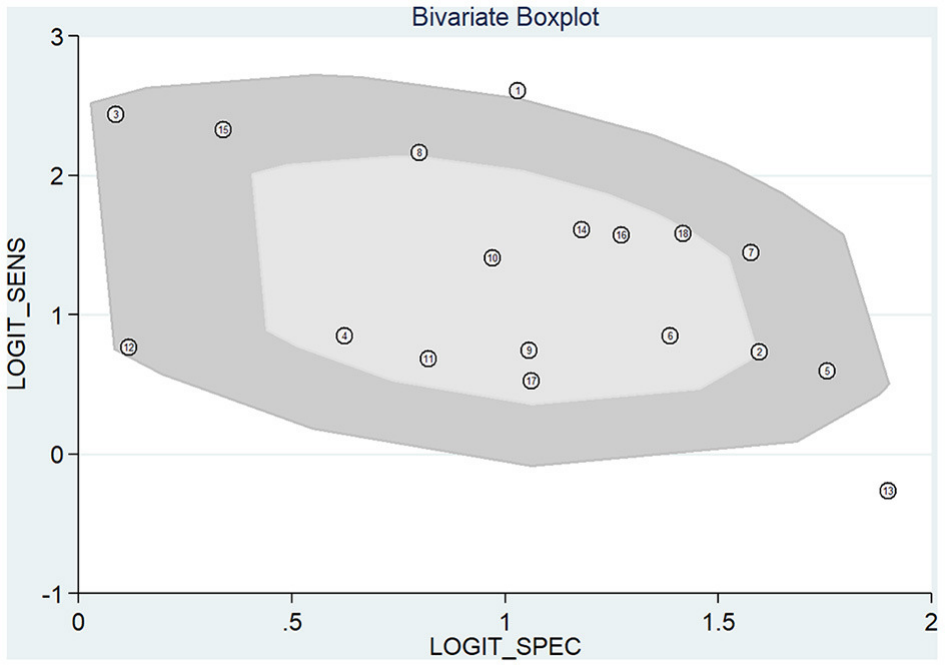
Bivariate boxplot evaluating the heterogeneity.

**Figure 5:**
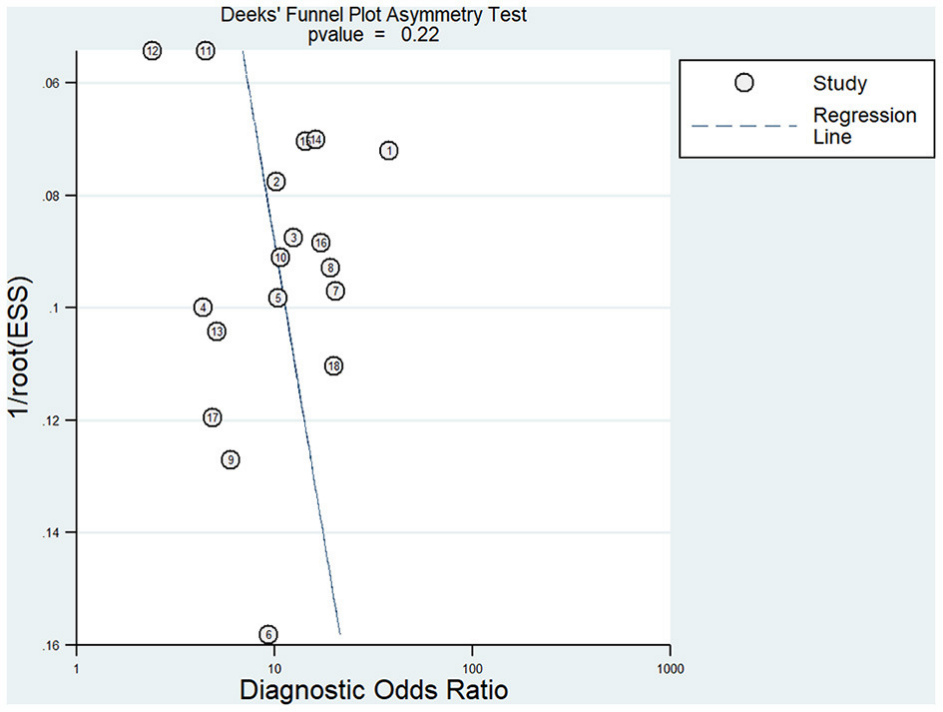
Deeks' funnel plot evaluating the potential publication bias.

Meta-analysis showed that the pooled sensitivity (Figure 3A), specificity (Figure 3B), DOR (Figure 3C), PLR (Figure 3D), NLR (Figure 3E), and AUC of the receiver operating characteristics (ROC; Figure 3F) curve, with a 95% CI, were 0.78 (0.71–0.83), 0.73 (0.68–0.78), 9.68 (6.76–13.85), 2.92 (2.45–3.50), 0.30 (0.23–0.39), and 0.810 (0.78–0.85), respectively. These results suggest a relatively moderate diagnostic value for circRNAs in discriminating CRC patients from normal individuals. There was significant heterogeneity among the included studies, as indicated by an I^2^ value of >50%. A bivariate boxplot was also constructed to evaluate the heterogeneity of each study (Figure 4). The funnel plot asymmetry test was applied to check for publication bias in the included studies, and the results, which were non-significant (*p* = 0.22), suggested that there was no potential publication bias between the studies (Figure 5).

### Subgroup Analysis

**Figure 6:**
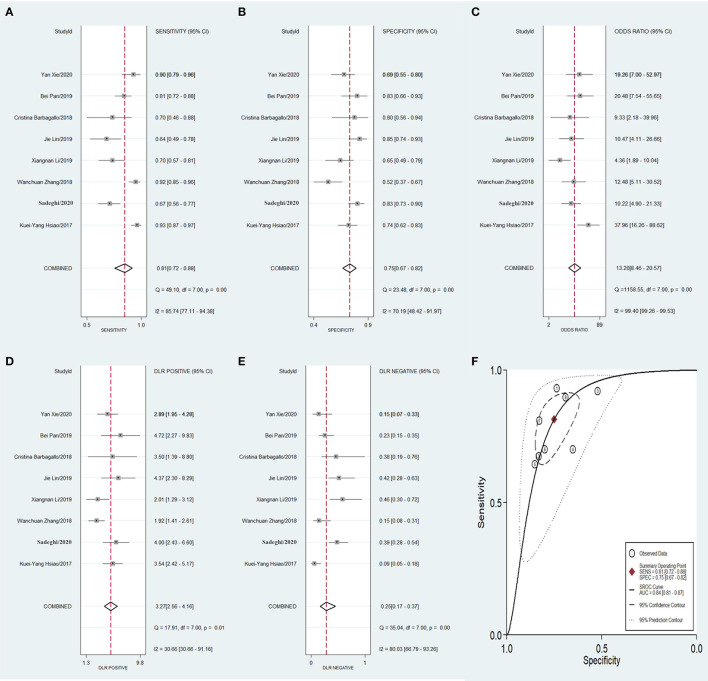
Forest plots of pooled diagnostic power of up-regulated circRNA as a biomarker for colorectal cancer, **(A)** Sensitivity, **(B)** Specificity; **(C)** DOR; **(D)** PLR; **(E)** NLR and **(F)** AUC. DOR, diagnostics odds ratio; PLR, positive likelihood ratio; NLR, negative likelihood ratio; AUC, the area under receiver operating characteristics curve.

**Figure 7:**
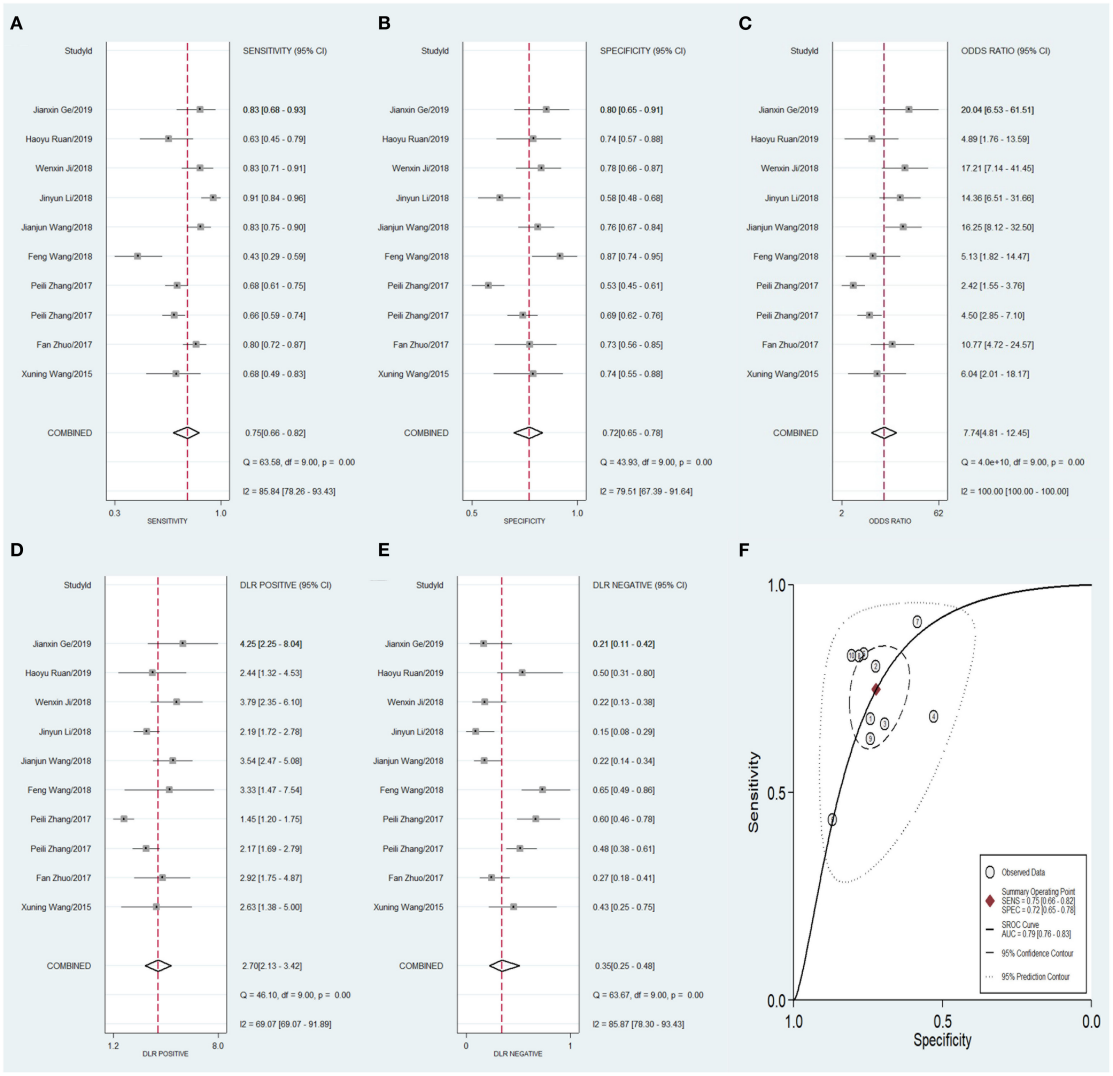
Forest plots of pooled diagnostic power of down-regulated circRNA as a biomarker for colorectal cancer, **(A)** Sensitivity, **(B)** Specificity; **(C)** DOR; **(D)** PLR; **(E)** NLR and **(F)** AUC. DOR, diagnostics odds ratio; PLR, positive likelihood ratio; NLR, negative likelihood ratio; AUC, the area under receiver operating characteristics curve.

To compare the diagnostic value between the different subgroups of circRNAs, including upregulated and downregulated circRNAs and tissue-derived and non-tissue-derived circRNAs, a stratified analysis was carried out to assess the diagnostic value of these groups. The pooled sensitivity (Figure 6A), specificity (Figure 6B), DOR (Figure 6C), PLR (Figure 6D), NLR (Figure 6E), and AUC of the summary receiver operating characteristics (sROC) curve (Figure 6F), with a 95% CI, for the upregulated circRNA group were 0.81 (0.72–0.88), 0.75 (0.67–0.82), 13.20 (8.46–20.57), 3.27 (2.56–4.16), 0.25 (0.17–0.37), and 0.84 (0.81–0.87), respectively, whereas the pooled sensitivity (Figure 7A), specificity (Figure 7B), DOR (Figure 7C), PLR (Figure 7D), NLR (Figure 7E), and AUC of the sROC curve (Figure 7F), with a 95% CI, for the downregulated group were 0.75 (0.66–0.82), 0.72 (0.65–0.78), 7.74 (4.81–12.45), 2.70 (2.13–3.42), 0.35 (0.25–0.48), and 0.79 (0.76–0.83), respectively. No publication bias was observed between the groups (*p* value of Deeks' Funnel Plot Asymmetry Test was 0.21 and 0.26, respectively). The results of the subgroup analysis suggested that upregulated circRNAs possess higher diagnostic power than the downregulated circRNAs.

**Figure 8:**
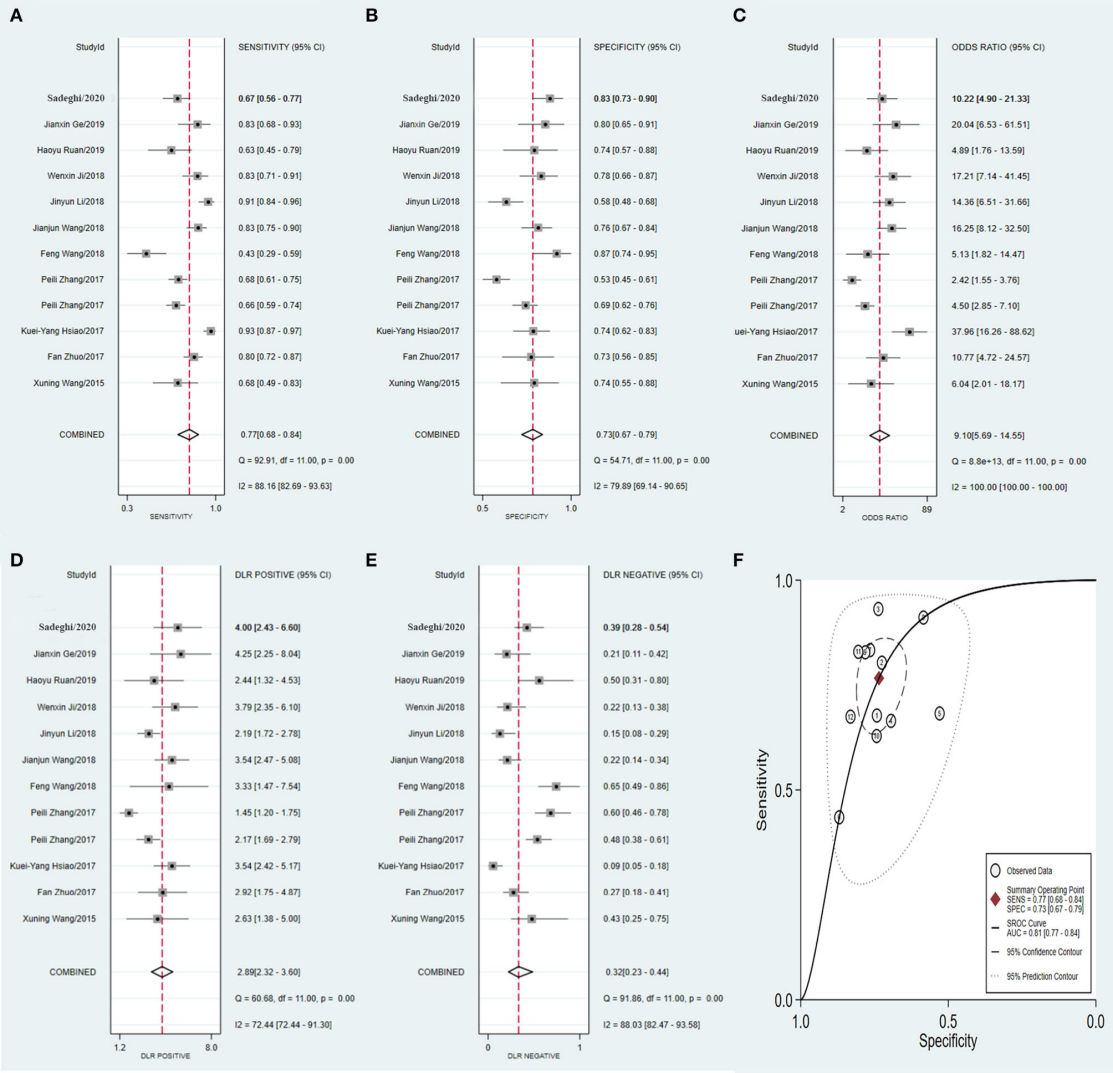
Forest plots of pooled diagnostic power of tissue-derived circRNA as a biomarker for colorectal cancer, **(A)** Sensitivity, **(B)** Specificity; **(C)** DOR; **(D)** PLR; **(E)** NLR and **(F)** AUC. DOR, diagnostics odds ratio; PLR, positive likelihood ratio; NLR, negative likelihood ratio; AUC, the area under receiver operating characteristics curve.

**Figure 9:**
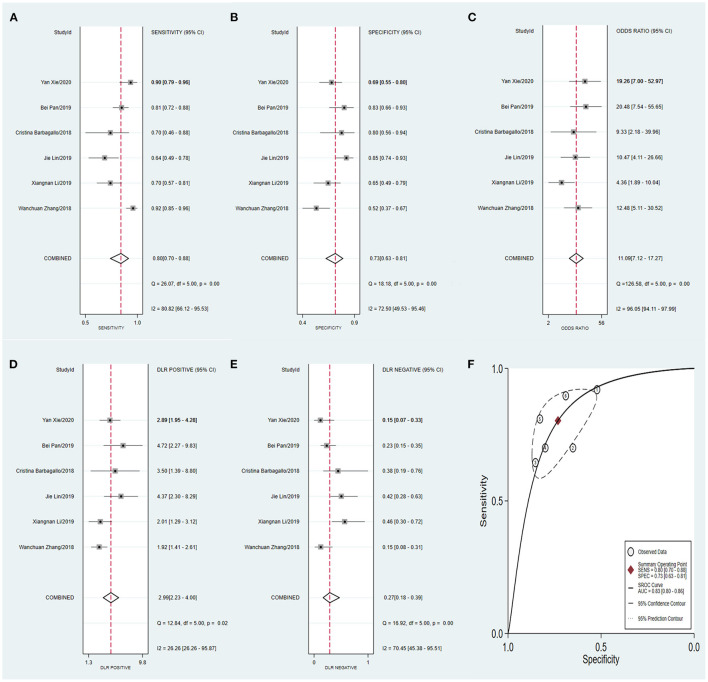
Forest plots of pooled diagnostic power of non-tissue-derived circRNA as a biomarker for colorectal cancer, **(A)** Sensitivity, **(B)** Specificity; **(C)** DOR; **(D)** PLR; **(E)** NLR and **(F)** AUC. DOR, diagnostics odds ratio; PLR, positive likelihood ratio; NLR, negative likelihood ratio; AUC, the area under receiver operating characteristics curve.

Similarly, the diagnostic values of the tissue-derived (Figure 8) and non-tissue-derived circRNAs (Figure 9) for CRC were also compared, but no significant difference was found between the two groups. However, as it is very convenient to collect the plasma of patients in the clinic, this may pave the way for effective diagnosis of early-stage CRC.

## Discussion

CRC is one of the most common malignancies and is ranked as the third-most (in both male and female) common cause of cancer-related death in America (33, 34). The incidence and mortality rates of CRC in the developing countries of Asia, such as China, have increased in recent years (35). Despite the extensive efforts made to define the typical signature of CRC to enable its early diagnosis, diagnostic markers with sufficient sensitivity and specificity to be applied in clinical practice have not been identified until date. CircRNAs are novel members of non-coding RNA family and are generated by the non-canonical splicing of linear pre-mRNA; they can regulate gene expression through binding proteins (36), acting as translation regulators (37), microRNA sponges (38) or RNA transporters (39). Dysregulated expression of circRNAs contributes significantly to cancer initiation and progression by regulating cancer cell growth, apoptosis, invasion, metastasis, and drug resistance (40). Compared with mRNA, whose half-life is only 10 h, the half-life of circRNA is longer, of more than 48 h (41). The high stability of circRNAs, owing to their circular structure, enables them to be more easily enriched in the human body, conferring superiority in terms of serving as novel diagnostic markers for CRC.

In this study, we conducted a meta-analysis, and 18 studies which explored the diagnostic accuracy of circRNAs for CRC were enrolled based on the inclusion/exclusion criteria. Several indices were used to assess the diagnostic value of circRNA for CRC, including sensitivity, specificity, PLR, NLR, DOR, and AUC. The value of DOR, which is a combination of sensitivity and specificity, showed a positive correlation with diagnostic performance. The pooled DOR of the included studies was 9.68 (6.76–13.85), suggesting the critical diagnostic value of circRNA in CRC patients. The overall performance of circRNAs was also evaluated using the AUC of the sROC. The pooled AUC was 0.81 (0.78–0.85), which represented the good diagnostic accuracy of circRNAs in distinguishing CRC patients from normal individuals. Low expression of circRNAs may affect the accuracy of detection (42), and then we analyzed the diagnostic power by subgroup analysis of different expression statuses. The results showed that upregulated circRNAs possess a much higher diagnostic power than downregulated circRNAs. However, no significant difference was found between the tissue-derived circRNAs group and non-tissue derived circRNAs group.

Our meta-analysis has some limitations. First, as almost all the included studies were from China, further studies are required to verify whether our conclusion is applicable to patients of different ethnicities. Second, data regarding individual circRNAs or combination strategies within the same studies (meaning that these data were collected from the same group of people) were collected and analyzed separately; this may play a negative role in the accuracy of the results as it may appear as if they were collected from several independent studies. Third, all the included studies used *GAPDH* as the reference gene, thus making it impossible to analyze the effect of the reference gene on diagnostic accuracy and to determine whether *GAPDH* is a good choice for the reference gene. Fourth, only a limited number of studies have assessed the diagnostic value of circRNAs using combination strategies. Therefore, further large-scale studies are needed to evaluate the diagnostic accuracy of circRNA as a diagnostic biomarker for CRC.

## Conclusion

The results of the meta-analysis carried out in this study suggested that circRNAs could potentially serve as diagnostic markers for CRC because of their ability to distinguish CRC patients from normal controls with relatively high sensitivity (0.78), specificity (0.73), and AUC (0.81). Further studies with larger sample sizes are needed to confirm this finding.

## Data Availability Statement

The original contributions presented in the study are included in the article/supplementary material, further inquiries can be directed to the corresponding author.

## Author Contributions

All authors had full access to all of the data in this present study and provided the guidance throughout the preparation of this manuscript.

## Conflict of Interest

The authors declare that the research was conducted in the absence of any commercial or financial relationships that could be construed as a potential conflict of interest.

## Publisher's Note

All claims expressed in this article are solely those of the authors and do not necessarily represent those of their affiliated organizations, or those of the publisher, the editors and the reviewers. Any product that may be evaluated in this article, or claim that may be made by its manufacturer, is not guaranteed or endorsed by the publisher.
